# Bruxism-Related Signs and Periodontal Disease: A Preliminary Study

**DOI:** 10.2174/1874210601812010400

**Published:** 2018-05-31

**Authors:** Rena Nakayama, Akira Nishiyama, Masahiko Shimada

**Affiliations:** Orofacial Pain Management, Oral Health Sciences, Graduate School of Medical and Dental Sciences, Tokyo Medical and Dental University, Tokyo, Japan

**Keywords:** Awake bruxism, Community periodontal index, Ischemia, Periodontal membrane, Questionnaire, Sleep bruxism

## Abstract

**Background:**

The effect of awake and sleep bruxism on periodontal disease has not been evaluated separately to date. Furthermore, there are few studies that have focused on awake bruxism with light force.

**Objective:**

The study aimed to investigate the frequency of sleep and awake bruxism in patients with periodontal disease.

**Methods:**

The subjects were 57 patients with periodontal disease who visited the Department of Periodontics of the Dental Hospital affiliated with Tokyo Medical and Dental University. Subjects were asked to fill out a questionnaire consisting of three items on bruxism (sleep and awake bruxism), and the maximum community periodontal index was investigated.

**Results:**

The proportions of individuals with high sleep bruxism-related signs and high awake bruxism-related signs were 6.0% and 44.0%, respectively. No significant difference was found in the comparison of maximum community periodontal index proportions between individuals with high sleep bruxism-related signs and high awake bruxism-related signs.

**Conclusion:**

The results of this survey of patients with periodontal disease showed that the proportion of subjects with high awake bruxism-related signs subjects was higher than those of the subjects with high sleep bruxism-related signs. Sleep bruxism has attracted attention as a factor influencing periodontal disease, and our data suggest that patients with periodontal disease demonstrate more bruxism while being awake than during sleep.

## 1 INTRODUCTION

Periodontal disease is characterized by gingival inflammation, periodontal pocket formation, bone loss, and clinical attachment loss [1]. It has been reported that subgingival biofilm plays a major role in the pathogenesis of periodontal disease by stimulating an immune response that can lead to periodontal breakdown [2-5]. The severity and progression of periodontal disease are influenced by environmental factors, in addition to genetic and acquired risk factors that can modify the host response [6].

Occlusal trauma is also an important factor in periodontal disease [7]. Occlusal trauma is a traumatic lesion occurring in periodontal tissues, of which there are two types. The first is primary occlusal trauma that is caused by strong non-physiological forces acting on healthy periodontal tissue, and the second is secondary occlusal trauma that is caused by normal force in tissue already weakened by periodontal disease. Bruxism is considered to be a non-physiological force.

Lobbezoo *et al*. have reported that “Bruxism is a repetitive jaw-muscle activity characterized by clenching or grinding of the teeth and/or by bracing or thrusting of the mandible. Bruxism has two distinct circadian manifestations: it can occur during sleep (indicated as sleep bruxism) or during wakefulness (indicated as awake bruxism)” [8].

Sleep Bruxism (SB) has been said to be associated with periodontal disease. SB cannot be considered as a direct cause of periodontal disease [9, 10]. However, when periodontal disease has already developed, symptoms progress rapidly due to the presence of SB, and it often progresses to severe periodontitis in a short period of time [11]. Therefore, SB is considered to be an exacerbating factor, rather than a causative factor, of periodontal disease, and night-guards have been used as a countermeasure.

In contrast, Awake Bruxism (AB) is associated with daytime clenching or diurnal clenching. Ferella **et al**. [12] measured the sustainable time of experimental clenching. In their study, it was reported that with Maximum Voluntary Clenching (MVC) set as 100%, the mean duration of sustained clenching was 1.4 minutes at 40% MVC and about 158 minutes at 7.5% MVC. Thus, a weak force can sustain the contact between the upper and lower teeth for a long time. It was reported that patients with Temporomandibular Disorders (TMD) considered the magnitude of the clenching force to be 70-80% [13]. Therefore, light forces may not be noticed as clenching.

There has been no report examining the effect of bruxism on periodontal disease separately for SB and AB. Furthermore, few studies have focused on AB with light force. In this study, we investigated the influence of SB and light force AB on periodontal disease.

## 2 MATERIALS AND METHODS

### 2.1 Study Subjects

The subjects of this study were first-visit patients, over 20 years old, who visited the Department of Periodontics at the Dental Hospital, Tokyo Medical and Dental University, between October 2014 and March 2015. The exclusion criteria were 1) the presence of TMD-related symptoms, 2) aggressive periodontitis, 3) periodontitis associated with systemic diseases, 4) the use of regular medications, such as anticonvulsants and calcium-channel blockers, and 5) the use of a removable partial denture.

This study was approved by the ethics committee of Tokyo Medical and Dental University (no. 974). The content of the study was explained to the 58 patients who satisfied the above criteria. All but one of these individuals signed a consent form (one person did not consent to participate in the study); thus, 57 patients eventually participated in this study. After participants had been diagnosed with periodontal disease by experts at the Department of Periodontics, the subjects completed a questionnaire on bruxism and underwent intraoral examination.

### 2.2 Study Details

Subjects were asked to fill out a questionnaire on bruxism, and underwent an examination of the oral cavity. The three questions about SB (Q1 and Q2) and light force AB (Q3) that formed part of the questionnaire are shown in Table **1**. For AB, the question was related to the presence of contact between the upper and lower teeth, regardless of the clenching force. All the questions were evaluated using a 5-point rating scale: 0) never, 1) hardly ever, 2) occasionally, 3) fairly often, 4) very often.

Furthermore, during the intraoral examination, the Community Periodontal Index (CPI) was measured by dentists. The CPI was defined by the World Health Organization (WHO) in 1997, as a modification of the CPI of treatment needs, which was developed collaboratively by the WHO and the Federation Dentaire Internationale in 1982. The assessments covered the following six teeth or pairs of teeth (Federation Dentaire Internationale two-digit notation): (i) 16 and 17; (ii) 11; (iii) 26 and 27; (iv) 36 and 37; (v) 31; and (vi) 46 and 47. For each tooth, the depth of the gingival pocket was measured using a WHO-type probe, and the evaluation was then coded as follows: 0: no signs; 1: hemorrhage present; 2: dental calculus present; 3: gingival pocket 4-5-mm depth; and 4: gingival pocket of at least 6-mm depth. For each of the pairs of molars, the higher code was taken as being representative, and the highest code among the six teeth or pairs of teeth was taken to be the individual patient’s highest code (maxCPI).

### 2.3 Statistical Analysis

Of the 57 respondents, seven had missing data and were excluded from the analysis. Finally, 50 subjects were included in the analysis. For Q1-3, participants with a score ≤ 2 (never, hardly ever, and occasionally) were assigned a value of “0 (low frequency),” whereas those with a score ≥ 3 (fairly often and very often) were assigned a value of “1 (high frequency).” When either of Q1 and Q2 scored 1, it indicated a strong possibility that the SB-Related Signs (SBRS) were excessive (high-SBRS). When Q3 was scored as 1, it indicated a strong possibility that the AB-Related Signs (ABRS) were excessive (high-ABRS). Both the numbers and ratio of each high-SBRS and high-ABRS were calculated, respectively.

Next, the proportions of individuals with high-SBRS and high-ABRS were calculated. For each bruxism type, maxCPI was compared between the risks (Mann-Whitney's U test). All statistical analyses were performed using *SPSS* version 21.0 software (IBM, Tokyo, Japan) and the level of significance was set at 0.05.

## 3 RESULTS

The mean age (standard deviation) of subjects was 55.4 ± 13.1 years. There were 32 females (64.0%). The number and proportions of each score for Q1-3 are shown in Table **2**. The proportions of individuals scoring “never” in Q1, Q2, and Q3 were 74.0%, 70.0%, and 24.0%, respectively. The proportion of individuals with high-SBRS and high-ABRS were 6.0% and 44.0%, respectively (Table **3**).

The results of CPI are shown in Table **4**. The total number of individuals with codes 3 and 4 was 41 (82.0%). No significant difference was found in a comparison of the maxCPI proportions between individuals with excessive signs related to each type of bruxism (Table **5**).

## 4 DISCUSSION

We investigated the influence of bruxism on patients with periodontal disease using questionnaires and intraoral examination. Our findings revealed that excessive ABRS is likely more common than excessive SBRS among these individuals.

The most reliable measurements of SB involve obtaining electromyograms of the masseter or temporal muscle, recording of facial parts on videotape, and recording of a grinding sound in addition to polysomnography during sleep [14]. However, since these evaluations require hospitalization, it is difficult to implement for many people over a short period of time. Therefore, EMG, clinical examination, and questionnaires are often used. The prevalence of SB differs depending on the measurement method used, but it is generally reported that about 10-13% of adults demonstrate SB [15]. In a previous population study of SB in a questionnaire survey of the general population, 8.6% answered SB as “regularly” and 13.7% answered “on occasion” for SB [16].

In this study, according to a questionnaire survey, three subjects (6.0%) had high-SBRS. This was a smaller percentage than that noted in the study by Khoury **et al**. [16]. Hanamura **et al**. [9] reported that there was no significant difference in tooth mobility and attachment level when comparing the relationship between SB and the severity of periodontal disease. Manfredini **et al**. [10] showed that SB is probably not a direct cause of damage to the periodontal tissue, because there is no clear relationship between SB and periodontal disease. Even in the present study, the association between SBRS and periodontal disease was not strong. However, if non-physiological mobility were present in the teeth, it is possible that a notable grinding sound may not be produced, due to the movement of the teeth during grinding, and the bed partner may not notice it. Therefore, objective measurement of SB levels, such as EMG measurements, will be necessary.

The AB considered in this study was not bruxism involving excessive force, but rather a minimal force. According to a study conducted on 2203 workers, using the same question items as in this study, the proportion of individuals with high-ABRS was about 20% [17]. In a study conducted on patients with painful TMD, using the same questionnaire, Sato *et al*. [18] reported that the proportion of individuals with high-ABRS was 52.4%. In the present study, 44.0% of individuals were judged as having high-ABRS. Although this proportion was slightly lower than that of individuals with painful TMD, it was more than twice as high as that in the general working population [17].

As described earlier, light-force clenching may be difficult to notice and can be maintained for a long time. In contrast, the total event time of SB per night is reported to be about 2 minutes for non-bruxers and about 10 minutes for bruxers [19]. Therefore, the total duration of SB is considered to be 15-20 minutes at the longest. Since the periodontal ligament membrane has viscoelastic properties, it can withstand short-term events involving a large bite force. However, in the case of a sustained, low-level force, the amount of deformation of the periodontal membrane increases with the passage of time, due to viscous drag, and capillary vessels in the periodontal membrane is compressed, causing ischemia [20]. Repetition of this ischemic condition may affect the resistance of the periodontal issue. In other words, it is possible that low-level, sustained force, such as AB, may cause greater damage to periodontal tissues than slightly stronger and shorter forces, such as SB.

Sato *et al*. [18] described awake sustained and low-level clenching as a Tooth Contacting Habit (TCH). At our facility, behavior modification is used to control TCH. Behavior modification is defined as the use of empirically demonstrated techniques to improve behavior. Behavior modification targets behaviors that can be objectively measured and aims to control these behaviors by reinforcing adaptive behavior and reducing maladaptive behavior. This process includes behavioral assessment to measure the target behavior and subsequent modifications to change that behavior. We propose that TCH can be effectively controlled using a time sampling method of behavioral assessment and a habit reversal method for behavior modification. This strategy includes three steps. The first step is a “motivation strategy,” wherein the patient confirms the habitual behavior using timers. The second step entails “awareness training” and “competing response training,” wherein the patient performs a substitute action in place of the adverse habitual behavior (for example, taking a deep breath) behavior immediately after noticing the inappropriate behavior through a reminder. After performing these tasks, the patient experiences less jaw muscle strain. The final step is “reinforcement”; the patient increases the frequency of noticing the behavior by performing the first and second steps repeatedly. It is possible for patients themselves to notice their own behaviors and to control them. As a result, the level of TCH can be expected to decrease, and it can be expected to reduce the burden of the force applied to the periodontal tissue.

This study had some limitations. Because this research was a preliminary survey-based study, the number of subjects was small (50 individuals), and all subjects had periodontitis. In future, studies using an equal number of healthy subjects (with no periodontal disease) as a control group are necessary. The designation of the severity of periodontal disease was moderately to severely biased. As the frequency of bruxism is also judged by questionnaires, investigations based on objective evaluation methods, such as electromyograms, are necessary in future.

## CONCLUSION

The results of this survey of patients with periodontal disease showed that the proportion of high-ABRS subjects was higher than those of high-SBRS subjects. SB has attracted attention as a factor influencing periodontal disease, but our results suggest that patients with periodontal disease demonstrate more bruxism while being awake than during sleep.

## Figures and Tables

**Table 1:** Questionnaire.

Q1	In the last 3 months, has it been pointed out to you that you make a tooth grinding sound during sleep?
Q2	Do you experience orofacial jaw muscle fatigue or pain when you are awake?
Q3	When you are concentrating on something, or during work, do your upper- and lower teeth contact each other?

All the questions were evaluated using a 5-point rating scale: 0) never, 1) hardly ever, 2) occasionally, 3) fairly often, and 4) very often.v

**Table 2:** Number and percentage of each evaluation in bruxism-related signs.

-	0. Never	1. Hardly Ever	2. Occasionally	3. Fairly Often	4. Very Often
**Q1**	37 (74.0%)	9 (18.0%)	3 (6.0%)	1 (2.0%)	0 (0%)
**Q2**	35 (70.0%)	5 (10.0%)	8 (16.0%)	1 (2.0%)	1 (2.0%)
**Q3**	12 (24.0%)	6 (12.0%)	10 (20.0%)	12 (24.0%)	10 (20.0%)

**Table 3:** Number and percentage of bruxism-related signs after binarizing the data.

-	Never Hardly Ever Occasionally	Fairly Often Very Often
	0	1
Q1	49 (98.0%)	1 (2.0%)
Q2	48 (96.0%)	2 (4.0%)
Q1 = 1 or Q2 = 1 (high-SBRS)	47 (94.0%)	3 (6.0%)
Q3 (high-ABRS)	38 (56.0%)	22 (44.0%)

SBRS, Sleep Bruxism-Related Signs; ABRS, Awake Bruxism-Related Signs

**Table 4:** Community periodontal index.

-	CPI (Median = 4.0, 25% = 3.0, 75% = 4.0)				
	0	1	2	3	4
N	1	3	5	11	30
%	2.0	6.0	10.0	22.0	60.0

CPI, Community Periodontal Index

**Table 5:** Relationship between bruxism-related signs and maximum Community Periodontal Index (CPI).

-		Maximum CPI	
		Median	*P*
High-SBRS	Negative (N = 47)	4.0	0.969
	Positive (N = 3)	4.0	
High-ABRS	Negative (N = 28)	4.0	0.991
	Positive (N = 22)	4.0	

SBRS, Sleep Bruxism-Related Signs; ABRS, Awake Bruxism-Related Signs; CPI, Community Periodontal Index
